# Antitumor Effects of Quercetin and Luteolin in A375 Cutaneous Melanoma Cell Line Are Mediated by Upregulation of P-ERK, c-Myc, and the Upstream GPER

**DOI:** 10.3390/life15030417

**Published:** 2025-03-07

**Authors:** Shaymaa A. Hussein, Nidaa A. Ababneh, Noor Tarawneh, Mohammad A. Ismail, Abdalla Awidi, Shtaywy Abdalla

**Affiliations:** 1 Department of Biological Sciences, School of Science, The University of Jordan, Amman 11942, Jordan; makishaymaa0077@gmail.com; 2 Cell Therapy Center, University of Jordan, Amman 11942, Jordan; n.ababneh@ju.edu.jo (N.A.A.); mohammad.ismail@adelaide.edu.au (M.A.I.); 3 Department of Pharmacy, Faculty of Pharmacy, Al-Zaytoonah University of Jordan, Amman 11733, Jordan; n.tarawneh@zuj.edu.jo; 4 South Australian ImmunoGENomics Cancer Institute, Adelaide Medical School, University of Adelaide, Adelaide, SA 5005, Australia; 5 Hemostasis and Thrombosis Laboratory, School of Medicine, The University of Jordan, Amman 11942, Jordan; 6 Department of Hematology and Oncology, Jordan University Hospital, The University of Jordan, Amman 11942, Jordan

**Keywords:** cutaneous melanoma, G-protein coupled estrogen receptor, GPER agonist, GPER antagonist, luteolin, quercetin

## Abstract

Cutaneous melanoma (CM) is the most aggressive and fatal malignancy among other skin cancers and its incidence has risen steadily recently around the world. Hormone-related therapy, particularly estrogen (E2) has been used as a prospective strategy for CM treatment. Quercetin and luteolin are flavonoids with antitumor effects against a wide range of cancers including CM. However, the underlying mechanism of their actions through GPER in CM is not fully understood. We examined the anti-tumor effects of quercetin and luteolin on the A375 CM cell line through activation of the G-protein coupled estrogen receptor (GPER). MTT assay was performed to assess the impact of flavonoids on cell viability. Apoptosis and cell cycle were studied by flow cytometry. Cell migration was evaluated by transwell assay. GPER expression and the effect of the flavonoids on the key signaling proteins were confirmed by immunofluorescence staining and Western blot, respectively. Results showed that quercetin and luteolin inhibited proliferation and migration, induced apoptosis, and blocked the cell cycle at S and G2/M in A375 cells. Immunofluorescence and immunoblotting data demonstrated the presence of GPER in this cell line and the two flavonoids enhanced its expression except at the high concentration of 100 µM. Quercetin and luteolin enhanced P-ERK and c-Myc expression, an effect abolished by the GPER antagonist G15, confirming GPER-mediated signaling. In conclusion, quercetin and luteolin exhibited anti-tumor effects on A375 melanoma cells via GPER activation, suggesting their potential as anti-melanoma therapeutics.

## 1. Introduction

Skin cancer is the most prevalent form of cancer worldwide, and it is expected to overtake heart disease as the leading cause of death in the coming decades [1]. It was reported that 97,160 new cases of skin cancer were diagnosed in the United States in 2023, which accounts for 5% of all cancer cases. Moreover, 7990 Americans lost their lives due to skin malignancies [2]. Depending on the cell type from which the disease evolved, skin cancer is classified as melanoma and non-melanoma skin cancer. Melanoma is a malignant tumor derived from the melanin-producing cells, the melanocytes. According to its origin, melanoma can be classified into cutaneous and non-cutaneous (uveal and mucosal) [3]. Cutaneous melanoma (CM) is the most aggressive and fatal malignancy among other skin cancers, and its incidence has risen steadily in recent decades around the world. This rapid increase is associated with a significant death rate and high healthcare costs. In 2020, CM accounted for about 325,000 (1.7%) cases of all diagnosed malignant cancers worldwide, with approximately 57,000 related fatalities [4]. Although CM is curable at early stages via surgical removal, advanced metastatic cutaneous melanomas can be challenging to treat and often resistant to therapies. Until a few years ago, advanced stages of the disease were exclusively treated with traditional chemotherapy (temozolomide and dacarbazine) and immunotherapy (interleukin-2 and interferon α-2b) [5]. Several challenges have been associated with the above treatments, such as poor response, rapid relapse, drug toxicity, adverse side effects, and unaffordable healthcare costs [6]. Therefore, searching for a new efficient therapeutic modality has drawn a lot of attention.

Estradiol (also known as 17β-estradiol) is the predominant and most active endogenous type of estrogens that regulates the function of reproductive organs in both sexes [7]. Moreover, E2 plays an important role in the growth and differentiation of normal tissues as well as different types of neoplasms, like breast, ovarian, endometrial, prostate, lung, kidney, pancreas, colon, brain, adrenals, and bone by binding to two specific estrogen receptors (ERs): ERα and ERβ which are members of the nuclear steroid hormone receptor superfamily [3].

Although controversial data were documented about the influence of E2 on CM, many researchers highlighted its ability to suppress tumor progression either through activation of ERβ or by receptor-independent mechanisms. In 2015, Marzagalli et al. reported that ERβ (but not ERα) is expressed in four types of melanoma cell lines (BLM, WM115, A375, WM1552) that have various genetic mutations, and it exhibits antitumor effects in BLM (NRAS-mutant) and WM115 (BRAF V600D-mutant) cells [8]. Therefore, hormone-related therapy has been taken into account as a promising strategy for CM treatment; it encouraged researchers to examine both endogenous and exogenous estrogens as potential drugs.

G protein-coupled estrogen receptor (GPER) is another important type of ER. It is a member of the G protein-coupled receptor family, which mediates rapid non-genomic effects of E2. GPER is widely expressed in numerous tissues like reproductive, nervous, bone, adipose, and digestive tissues [9]. Activation of GPER by its specific ligand (E2) triggers several intracellular pathways, such as activation of adenylyl cyclase (AC), MAPK, PI3K/Akt, and trans-activation of epidermal growth factor receptor (EGFR). Recently, numerous studies [10,11] have been carried out to investigate the role of GPER activation in the development of different cancer types, mainly breast, ovarian, endometrial, and prostate cancers. Controversial results suggested that the effect of GPER varies according to tumor type, site, stage, and environment [12]. In addition to E2, which is the main endogenous ligand for GPER with a binding affinity of about 3–6 nM, a number of GPER ligands with either agonistic or antagonistic effects were identified [13]. These include other forms of estrogens, such as 17-α estradiol, estrone, estriol (E3), and other compounds [14]. The presence of selective pharmacological ligands (agonists or antagonists that bind GPER, but not ER α/β) was fundamental to clarify the physiological role of GPER. In 2006, the first GPER selective agonist, G-1, was identified [15]. It has a stronger binding affinity for GPER than E2, and it was widely used by researchers to examine the biological effects of GPER in health and disease states and to discover new potential ligands. Subsequently, the two GPER selective antagonists, G15 and G36, were synthesized in 2009 and 2011, respectively [16].

Phytoestrogens are secondary metabolites produced by plants under environmental stresses and can be classified into two main groups: flavonoids (isoflavones, coumestans, and prenyl flavonoids) and non-flavonoids (mainly lignans). Due to their structural similarity with E2, phytoestrogens can interact with ERs (both classical and GPER) and exert both estrogenic and/or anti-estrogenic effects [17]. Flavonoids are natural polyphenolic compounds present in certain fruits and vegetables [18]. Quercetin (3, 3′, 4′, 5, 7-pentahydroxyflavone) is the main flavonoid in the human diet and is characterized by the presence of five hydroxyl groups (-OH) that are found on the C6-C3-C6 backbone structure, especially a 3-OH group on the pyran ring [19,20]. Quercetin acts as an anticancer agent by several mechanisms, such as cell cycle arrest, inhibition of MAPKs and Akt pathways, preventing cell proliferation as well as apoptosis induction [21,22]. It was demonstrated that quercetin inhibited proliferation and induced apoptosis of murine B16 melanoma cell lines in vitro [23]. Similarly, quercetin showed anti-melanoma effects both in vitro and in vivo, and increased apoptosis of A375SM human melanoma cell lines through activation of JNK/P38 MAPK signaling pathway [24]. Regarding the phytoestrogen properties, quercetin can bind to GPER as a natural agonist as demonstrated by Maggiolini, et al. (2004). In their study, GPER in SKBR3 breast cancer cells was activated by quercetin leading to rapid activation of ERK1/2 and upregulation of c-fos [25]. Within the same context, it was shown that quercetin suppressed osteoclastogenesis by GPER activation and inhibition of Akt phosphorylation [26].

Another important flavonoid is luteolin (3′,4′,5,7-tetrahydroxy flavone), which is characterized by the presence of a double bond between C2 and C3 and two hydroxyl groups in each benzene ring. As an anti-cancer agent, this compound can suppress cell transformation, angiogenesis, metastasis, and invasion of cancer cells via different mechanisms including cell cycle regulation, inhibition of kinases, stimulation of apoptosis, and alteration of gene expression [27]. In melanoma, luteolin was able to block the tumor progression in the murine B16F10 melanoma cell line, and to reduce the invasiveness by targeting β3 integrin and epithelial-mesenchymal transition [28]. In another study on human choroidal melanoma, it was demonstrated that luteolin decreased the expression of vascular endothelial growth factor (VEGF) and induced apoptosis and cell cycle arrest in C918 and OCM-1 cells [29]. Little information is available about the interaction of luteolin with ERs. Molecular docking studies suggested that luteolin has the ability to bind to ERα and ERβ as well as to GPER [30].

The aim of the present study was to investigate the anti-tumor effects of quercetin and luteolin on GPER-expressing cutaneous melanoma cell line, A375. We evaluated the potential agonistic properties of quercetin and luteolin as phytoestrogens by exploring their ability to activate GPER, along with their influence on the key signaling pathways Ras/Raf/Erk and PI3K/Akt. The data shows that quercetin and luteolin increased P-ERK and c-Myc and these effects were reversed by the specific antagonist of GPER, indicating that the flavonoids’ effects were mediated by GPER, highlighting the potential anti-melanoma therapeutic effects of the two compounds.

## 2. Materials and Methods

### 2.1. Cell Line and Culture Conditions

A375 melanoma cell line (Cat no.300110) was purchased from Cell Line Service (CLS, GmbH, Eppellheim, Germany). It is a primary cutaneous melanoma isolated from the skin of a 54-year-old female patient with malignant melanoma, and it has an epithelial-like morphology. A375 cell harbors the BRAF V600E mutation, and it was chosen in the present study because it expresses the protein of interest, G protein-coupled estrogen receptor (GPER) [31]. Cells were cultured in a minimum essential medium (MEM) (Euro Clone, Milan, Italy) supplemented with 10% fetal bovine serum (FBS), 1% of 100× antibiotic/antimycotic mixture (Gibco, Waltham, MA, USA), 1% of 100× GlutaMAX (Gibco, Waltham, MA, USA) and 1% of 100× MEM non-essential amino acids (EuroClone, Milan, Italy). Cells were maintained in a CO_2_ incubator (Thermo Scientific, Waltham, MA, USA) under standard growth conditions at 37 °C, 5% CO_2,_ and 95% humidity and were routinely tested for mycoplasma.

### 2.2. Chemicals and Preparation

Quercetin (more than 98% purity) was purchased from Santa Cruz Biotechnology, Dallas, TX, USA, luteolin (more than 98% purity) from Biosynth, Irvine, CA, USA, G1 (GPER-selective agonist) [32] and G15 (GPER-selective antagonist) [33] from Tocris Bioscience, Oxford, UK. Stock solutions (50 mM) were made by dissolving the proper amount of compounds in dimethyl sulfoxide (DMSO) (Fisher Scientific, Waltham, MA, USA), and stored at −20 °C. The desired final concentrations were freshly prepared by diluting stock solutions in culture media at the beginning of each experiment. Final DMSO concentration was 0.2% in all working solutions.

### 2.3. Cell Viability Assay

The effect of treatments on cell viability and growth was examined by MTT (3-(4,5-dimethylthiazol-2-yl)-2,5-diphenyltetrazolium bromide) assay [34,35] with some modifications. Briefly, 7 × 10^3^ cells/well of A375 were suspended in 100 μL/well of culture media and seeded in 96-well plates. After 24 h of incubation, adherent cells were treated with 1, 3, 10, 30, and 100 μM of quercetin or luteolin as well as 1 μM of G1 (a positive control). Cells treated with 0.2% DMSO (vehicle) served as a negative control. After 24 h of incubation, culture medium in each well was replaced by a fresh medium containing 10 µL of MTT solution (Promega, Madison, WI, USA) and cells were incubated for 3 h at 37 °C. A 100 µL amount of the solubilization solution/stop mix (Promega, Madison, WI, USA) was then added to each well and kept in dark overnight to ensure complete dissolution of the generated formazan crystals. In another set of experiments, A375 cells were pretreated with 3 μM of GPER antagonist G15 for 24 h then cells were treated with the most effective concentrations (10, 30, and 100 µM) of quercetin or luteolin, or 1 µM of G-1 for 48 h The cells were then processed similarly.

Absorbance was measured at 570 nm using Biotek Cytation 5 microplate reader (Agilent, Santa Clara, CA, USA), and the same procedure was repeated after 48 and 72 h The percentage of cell viability was calculated as follows:

$$\left(\%\right)cell\;viability=\frac{\left(Optical\;density\;of\;treated\;sample\right)}{\left(Optical\;density\;of\;control\right)}\times 100$$

Values of the 50% inhibitory concentrations (IC_50_) were determined using Graph Pad prism software, version 8.

### 2.4. Cell Morphology

A375 cells were seeded in 6-well plates at a density of 2.5 × 10^5^ cells/well for 24 h to allow cell adhesion. Cells were then incubated with quercetin or luteolin (1, 3, 10, 30, and 100 µM), G1 (1 µM), and 0.2% DMSO (vehicle) for 48 h. Changes in cell morphology were observed and photographed using EVOS XL, core inverted microscope (Invitrogen, Waltham, MA, USA).

### 2.5. Flow Cytometry

#### 2.5.1. Apoptosis Assay

A375 cell line cells were grown in 6-well plates at a density of 2.5 × 10^5^ cells/well and allowed to adhere for 24 h under standard growth conditions. Cells were subjected to 1, 3, 10, 30, and 100 µM of quercetin or luteolin, and to 1 µM G1 or 0.2% DMSO. After incubation for 48 h, culture media were aspirated, and the treated cells were detached using trypsin. Cells were then washed with 1 mL/well PBS, harvested, and transferred into 5 mL flow cytometer tubes. FACS^®^ Annexin V-FITC Apoptosis Detection Kit (R&D systems, Minneapolis, MN, USA) was used to investigate cell apoptosis. Following manufacturer’s instructions, cell suspensions were centrifuged for 5 min at 400× *g*. The supernatants were decanted and 100 μL/sample of the incubation reagent solution (10× binding buffer 10 μL, Propidium Iodide (PI) 10 μL, TACS Annexin V-FITC 1 μL, and distilled water 79 μL) were added to cell pellets and incubated for 15 min in the dark. Stained cells were then examined using BD FACS Canto II flow cytometer. A total of 10,000 events from each sample were counted and sorted out based on fluorescence intensity into 4 groups: viable (Annexin V−/PI−), early apoptotic (Annexin V+/PI−), late apoptotic (Annexin V+/PI+) and necrotic (Annexin V−/PI+). The percentage of cells in each group was quantified and analyzed using Flow Jo software, version 10 and data were presented as dot plots [36].

#### 2.5.2. Cell Cycle Assay

Then, 2.5 × 10^5^ cells/well were cultured in 6-well plates. After 24 h of incubation, adherent cells were exposed to quercetin or luteolin (1, 3, 10, 30, and 100 µM), G1 (1 µM), and 0.2% DMSO for 48 h. Tali cell cycle kit (Invitrogen, Waltham, MA, USA) was used according to manufacturer’s instructions. In brief, cells were trypsinized, harvested, transferred into 15 mL conical tubes, and centrifuged at 400× *g* for 5 min. The supernatant was discarded, and cells were fixed by adding 400 μL of cold PBS and 800 μL of cold ethanol (70%) for each pellet and vortexed, then incubated overnight at −20 °C. Cells were then centrifuged, the supernatant was decanted, and cells were washed twice with PBS prior to transfer into flow cytometer tubes. Finally, cells were centrifuged and incubated with 200 μL of staining solution (PI and RNase) for 40 min at 37 °C. A total of 10,000 events per sample were counted and PI intensity was measured using BD FACS Canto II flow cytometer. Percentage of cells in G1, S, and G2/M phases was calculated based on DNA content. Data were analyzed by Flow Jo software and illustrated as histograms [37].

### 2.6. Transwell Migration Assay

The transwell assay was carried out to examine the potential effects of tested treatments on migration of melanoma cell lines. A375 cells were routinely cultured in 6-well plates at a density of 5 × 10^5^ cells/well. After 24 h, cells were treated with 10, 30, and 100 µM of quercetin or luteolin, 1 µM of G1, and 0.2% DMSO for 48 h. To assess the role of GPER, cells were exposed to 3 µM of GPER-specific antagonist, G15, for 24 h before treatment with the tested flavonoids. Treated cells were then trypsinized and counted. Then, 1 × 10^5^ cells/well were suspended in 200 μL serum-free MEM and seeded into the upper chamber of 24-well standing SPLInsert™ plate, with 8 μm pore size membrane (SPL Life Sciences, Pocheon-si, Republic of Korea). A 750 μL amount of complete medium with 10% FBS (as chemoattractant) was added to the lower chamber. The plates were incubated at 37 °C for 48 h to allow cell migration. Next, the medium on the upper chamber was aspirated and cotton swabs were used to remove non-migrated cells from the upper surface of the membrane. Migrated cells that adhered to the lower surface of the membrane were fixed with 4% paraformaldehyde for 20 min and stained with 0.3% crystal violet for 20 min before being imaged by EVOS XL core inverted microscope at 10× objective magnification. The number of migrated cells was counted in five randomly selected fields [38].

### 2.7. Immunofluorescence (IF) Assay

The expression of GPER was examined by immunofluorescence assay. A375 cells were grown on sterile coverslips in 12-well plates at a density of 1 × 10^5^ cells/well for 24 h. Cells were treated with 10, 30, and 100 µM of quercetin or luteolin, 1 µM of G1, and 0.2% DMSO (vehicle) for 48 h, then washed with PBS and fixed for 15 min with 4% paraformaldehyde (250 µL per well). Cells were then washed 3 times with PBS and incubated for 1 h with 500 µL/well of blocking solution (2.5% bovine serum albumin (BSA), 2.5% normal goat serum (NGS), and 0.5% Triton-X100, all dissolved in PBS). After 3 times washing with PBS, cells were stained with 250 µL/sample of anti-GPER rabbit primary antibody (1:1500) (ES 11471 ELK Biotechnology, Denver, CO, USA) in blocking solution and incubated overnight at 4 °C with shaking. The next day, cells were rinsed with PBS, and 300 µL/well of Alexa Fluor 488 goat anti-rabbit IgG secondary antibody (1:500) (A32731, Invitrogen, Waltham, MA, USA) in blocking solution was added to each well and incubated for 2 h at room temperature. For nuclear staining, 4′, 6-diamidino-2-phenylindole dihydrochloride (DAPI) (62248, Thermo Scientific, Waltham, MA, USA) was diluted to 1:1500 in PBS and 500 µL was added to each well for 5 min at room temperature. Cells were further washed with PBS and coverslips were placed onto glass slides using anti-fade mounting medium (Abcam, Cambridge, UK), and photographed by time-lapse fluorescence microscope (Carl Zeiss, Jena, Germany). Images were analyzed by cell profiler software, version 3 and means of fluorescence intensity (MFI) were calculated [39].

### 2.8. Immunoblotting Assay

Immunoblotting analysis was performed to assess the influence of tested flavonoids on GPER expression and the related signaling pathways (Ras/Raf/Erk and PI3K/Akt). Cells were cultured in T25 tissue culture flasks at a density of 1 × 10^6^ cells/flask and incubated at 37 °C until 85–90% confluence. Cells were then subjected to 10, 30, and 100 µM of quercetin or luteolin, 1 µM of G1 or 0.2% DMSO for 24 h In order to determine the involvement of GPER, cells were pre-treated for 24 h with 3 µM of the inhibitor G15. RIPA lysis buffer (SC-24948A; Santa Cruz Biotechnology, Dallas, TX, USA) supplemented with phosphatase inhibitor cocktail IV (ab201115; Abcam, Cambridge, UK) was used for the extraction of total proteins and lysates preparation. The concentration of total protein in samples was measured using the bicinchoninic acid protein assay kit (SMART BCA, INtRON biotechnology, Seoul, South Korea) according to manufacturer instructions. The equivalent of 20 µg/lane of protein lysates were loaded and separated on 8% SDS-PAGE before being transferred onto nitrocellulose membranes. An amount of 3% BSA (Biowest, PA0005R023, Leon, France) tris-buffered saline (TBS) was used to block the membranes overnight at room temperature. Target proteins were detected by overnight incubation at 4 °C with the specific primary antibodies listed in Table 1. After washing with TBS buffer, membranes were incubated with horseradish peroxidase (HRP) conjugated goat anti-rabbit (AB6721, Abcam, Cambridge, UK; 1:1000) or HRP-conjugated goat anti-mouse (AB97023, Abcam, Cambridge, UK; 1:1000) secondary antibodies for 1 h. Protein bands were detected by chemiluminescence method using 4-chloro-1-naphthol substrate (BioWorld 40300004-1, Visalia, CA, USA). Membranes were imaged with a ChemiDocTM XRS+ System (Bio-Rad, Hercules, CA, USA) and densitometry analysis was performed using Image Lab 6 software (Bio-Rad).

### 2.9. Statistical Analysis

Results were statistically analyzed using GraphPad Prism software, version 8. Data were expressed as the means ± SD. Statistical significance of apoptosis and cell cycle data were analyzed using two-way ANOVA, followed by Dunnett’s multiple comparisons test. MTT, immunofluorescence, transwell migration, and Western blot data were analyzed using one-way ANOVA followed by Dunnett’s multiple comparisons test. Statistical significance was declared when *p* was <0.05 or had lower values as indicated in each figure legend.

## 3. Results

### 3.1. Cell Viability 

**Figure 1:**
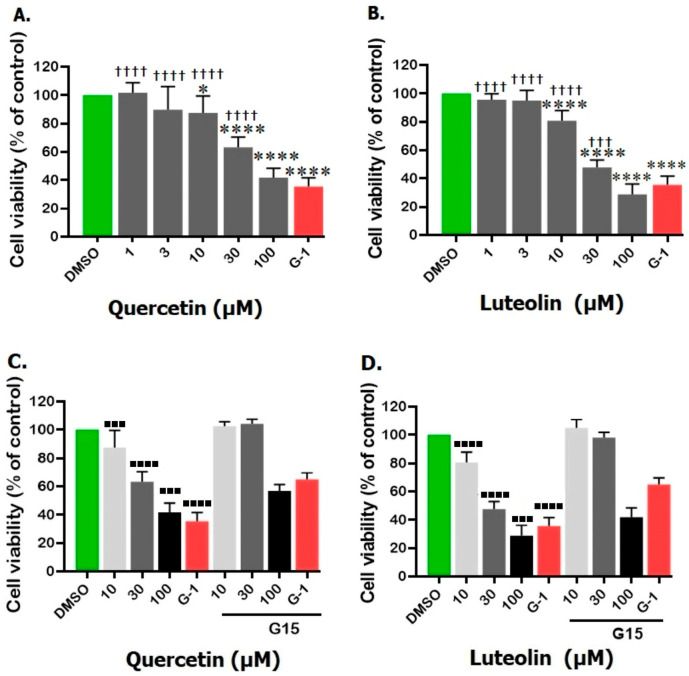
Effects of the tested flavonoids on proliferation of A375 CM cells. Cells were treated for 48 h with 1, 3, 10, 30, and 100 µM of quercetin or luteolin; G-1 (1 µM) served as a positive control and DMSO (0.2%) as a negative control. Percentage of cell viability was calculated (**A**,**B**). Pretreatment with G15 significantly reversed the inhibitory effects of quercetin, luteolin, and G-1 on cell viability (**C**,**D**). Data are presented as the means ± SD of three independent experiments. Statistical analysis was performed using one-way ANOVA followed by Dunnett’s multiple comparisons test. (* *p* < 0.05, **** *p* < 0.0001 compared to DMSO; ††† *p* < 0.001, †††† *p* < 0.0001 compared to G-1; ■■■ *p* < 0.001, ■■■■ *p* < 0.0001 compared to the indicated concentrations of quercetin, luteolin, or G-1 in the presence of G15).

Quercetin and luteolin decreased cell viability in a dose-dependent manner compared to the control (DMSO). The two flavonoids exerted the highest inhibitory effects after 48 h and there was no further inhibition when the incubation period was extended to 72 h (the maximum percentage of cell viability was 34.9 ± 1.3 for quercetin and 22.9 ± 0.8 for luteolin after 72 h of incubation). Therefore, a 48 h incubation period was chosen in all the following experiments. No significant changes in A375 cell proliferation were noticed at the lowest concentrations (1 and 3 µM) of quercetin or luteolin as compared to the negative control (DMSO) after 48 h. At higher concentrations (10, 30, and 100 µM), cell viability was significantly reduced (Figure 1A,B). The estimated IC50 values were 38.6 µM for quercetin and 19.6 µM for luteolin. In addition, G-1 (positive control) at a concentration of 1 µM exhibited anti-proliferative effects against A375 cells and significantly decreased their viability by 35.4 ± 1.2% relative to DMSO-treated cells. Pretreatment of A375 with 3 µM G15 reversed the antiproliferative effects induced by 10, 30, and 100 µM of quercetin or luteolin, and those by G-1, and significantly increased cell viability (Figure 1C,D).

### 3.2. Morphological Changes in Melanoma Cell Lines

**Figure 2:**
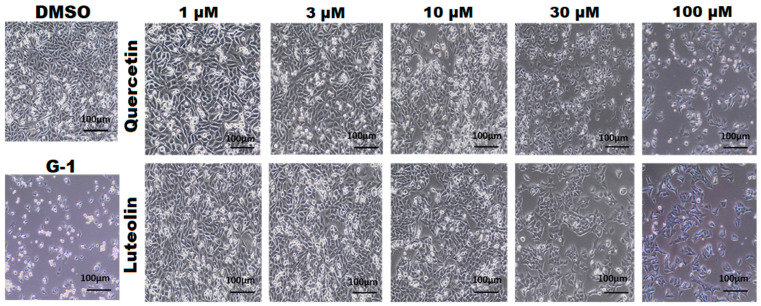
Effects of quercetin and luteolin on the growth and morphology of A375 CM cells. Cells were treated with DMSO (0.2%) as a negative control and G-1 as a positive control, and with the indicated concentrations of quercetin (**upper panel**) or luteolin (**lower panel**).

As shown in Figure 2, DMSO-treated A375 cells appeared confluent, well attached to the tissue culture plate, and displayed typical epithelial-like morphology with a polygonal shape. Notable decreases in cell confluence and floating detached cells were observed following treatment with the GPER agonist, G-1 (the positive control). Cells exposed to low concentrations (1 and 3 µM) of quercetin or luteolin maintained their morphology, but at higher concentrations (10, 30, and 100 µM) cells were less confluent and some of them had detached from the culture plate.

### 3.3. Flowcytometry

#### 3.3.1. Apoptosis/Necrosis Quantification

**Figure 3:**
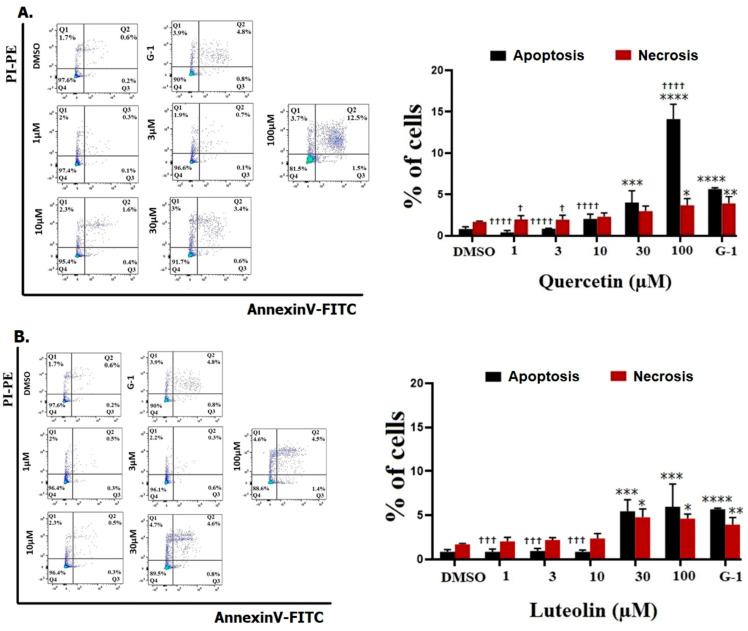
Flow cytometry analysis of cell apoptosis. A375 cells were treated with DMSO (0.2%) as a negative control and G-1 (1 µM) as a positive control, quercetin (**A**) or luteolin (**B**) at the indicated concentrations. After 48 h, treated cells were stained with Annexin V/PI, and cell apoptosis was determined using flow cytometry. Representative flow cytometry plots are shown (left panels). Data were analyzed using FlowJo software, version 10 (right panels). Results are presented as the means ± SD of three independent experiments. Statistical significance was determined using 2-way ANOVA followed by Dunnett’s multiple comparisons test. (* *p* < 0.05, ** *p* < 0.01, *** *p* < 0.001, **** *p* < 0.0001 compared to DMSO; † *p* < 0.05, ††† *p* < 0.001, †††† *p* < 0.0001 compared to G-1).

The ability of tested flavonoids to induce apoptosis/necrosis in A375 CM cells was evaluated after 48 h. No significant increase in the rate of cell apoptosis and necrosis was found after exposure to 1, 3, and 10 µM of quercetin or luteolin. When the concentration of quercetin was increased to 100 µM, there was a significant increase in both apoptosis (14.1%) and necrosis (3.7%) (Figure 3A). Similarly, higher concentrations of luteolin (30 and 100 µM) caused a significant increase in cell apoptosis to 5.4% and 5.9%, as well as cell necrosis to 4.7% and 4.6%, respectively (Figure 3B). A 1 µM amount of G-1 caused an increase in cell apoptosis to 5.6% and cell necrosis to 3.9%.

#### 3.3.2. Cell Cycle Analysis

**Figure 4:**
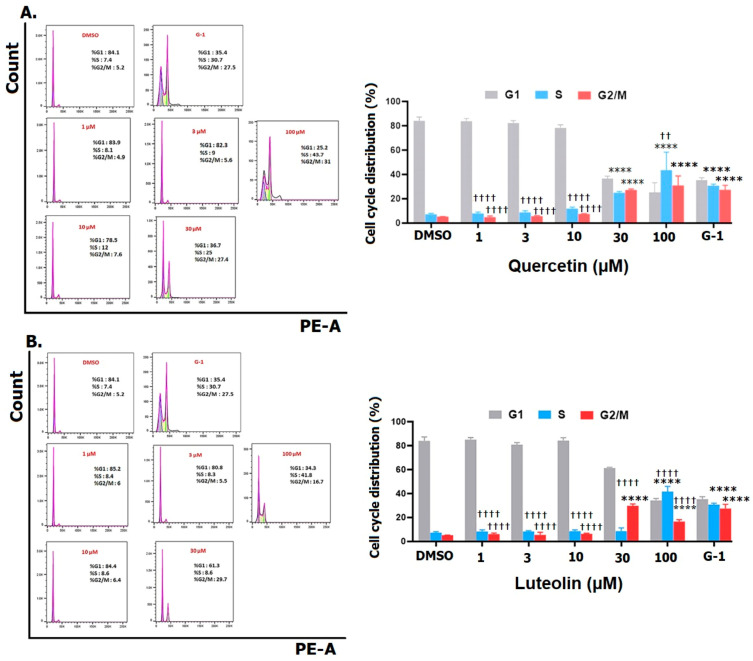
Cell cycle distribution of A375 CM cell lines. Cells were treated with the indicated concentrations of quercetin (**A**) or luteolin (**B**), A 1 µM of G-1 as a positive control, and 0.2% DMSO as a negative control. After 48 h, cells were fixed, stained with PI, and analyzed flow cytometry (right panels). The percentage of cells in G1, S, and G2/M phases for each treatment was calculated and analyzed (left panels). Data are presented as the means ± SD of three independent experiments. Statistical significance was determined using 2-way ANOVA followed by Dunnett’s multiple comparisons test. (**** *p* < 0.0001 compared to DMSO; †† *p* < 0.01, †††† *p* < 0.0001 compared to G-1).

Exposure of A375 cells to 30 µM of quercetin caused a significant increase in the proportion of S and G2/M phase, which peaked at 100 µM to reach 43.7% for the S phase and 31% for the G2/M phase (Figure 4A). A maximum increase in the proportion of G2/M to 29.7% was observed following treatment with 30 µM of luteolin, while the maximum increase of 41.8% in the S phase was reached after 100 µM (Figure 4B). Furthermore, when cells were treated with G-1, a significant rise in the percentage of cells in the S phase to 30.7% was noted, accompanied by a significant increase in the G2/M phase to 27.5%.

### 3.4. Migration of Melanoma Cells

**Figure 5:**
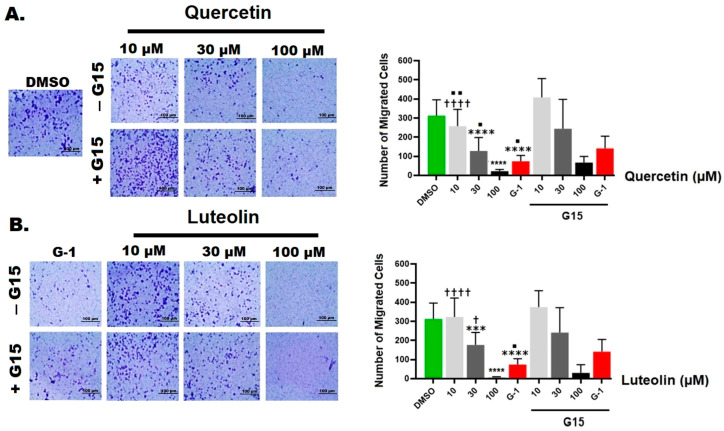
Migration of A375 cells under different treatments. Cells were exposed for 48 h to the indicated concentrations of quercetin (**A**) or luteolin (**B**), G-1 (positive control), and 0.2% DMSO (negative control), with or without 3 µM of G15 (GPER antagonist). Transwell migration assay was performed for 48 h and the number of migrating cells is shown in the right panels. Values are means ± SD of 10 fields/treatment. Statistical significance was determined using one-way ANOVA followed by Dunnett’s multiple comparisons test. (*** *p* < 0.001, **** *p* < 0.0001 compared to DMSO; † *p* < 0.05, †††† *p* < 0.0001 compared to G-1; ■ *p* < 0.05, ■■ *p* < 0.01compared to treatment with G15).

The effects of quercetin and luteolin on the migration ability of A375 were assessed by transwell migration assay. The number of migrated cells significantly decreased to 73 cells/field after treatment with G-1, compared to DMSO control (312 cells/field). Treatment with 10, 30, or 100 µM quercetin suppressed the migration in a concentration-dependent manner, and the number of migrated cells per field decreased to a minimum of 21 cells/field at 100 µM (Figure 5A). On the other hand, cell migration was not affected by the presence of 10 µM of luteolin, whereas at 30 and 100 µM, the number of migrated cells was significantly lower compared to the control (176 and 6 cells/field, respectively (Figure 5B). The anti-migratory effects induced by quercetin and G-1 were only partially reversed when A375 cells were pre-treated with the GPER antagonist G15.

### 3.5. Immunofluorescence (IF) Staining of GPER

**Figure 6:**
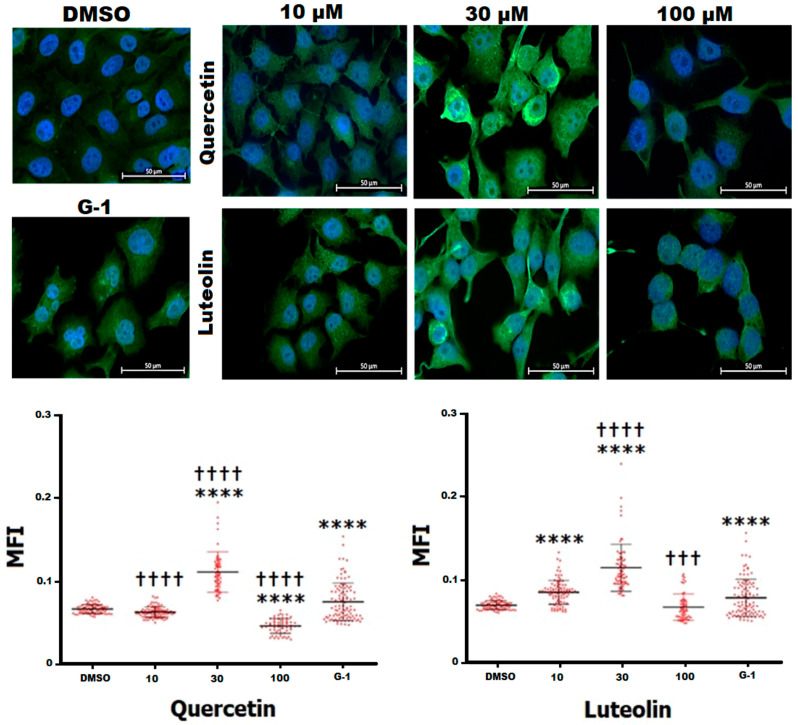
Detection of GPER expression in A375 melanoma cells by immunofluorescence staining. Cells were treated for 48 h with 10, 30, and 100 µM of quercetin, luteolin, or 1 µM G-1 as a positive control. DMSO (0.2%) was used as negative control. Representative immunofluorescence images captured by time-lapse fluorescence microscope are shown (upper panels). Green signal indicates positive staining for GPER, while the blue signal (DAPI) represents the nuclei. Scatter dot plots show the mean fluorescence intensity (MFI) for GPER determined by cell profiler software (lower panel). Statistical significance was determined using one-way ANOVA followed by Dunnett’s multiple comparisons test (**** *p* < 0.0001 compared to DMSO; ††† *p* < 0.001, †††† *p* < 0.0001 compared to G-1).

To confirm the expression of GPER, immunofluorescence staining was performed. A375 cells stained immunopositive for GPER (Figure 6). To further investigate whether the tested flavonoids can modulate the expression of GPER, mean fluorescence intensity (MFI) was calculated. DMSO-treated cells had an MFI of 0.066. Exposure to 30 µM of quercetin significantly increased the immunostaining of GPER (MFI = 0.11), while at 100 µM significant decrease in MFI to 0.04 was observed compared to DMSO control. On the other hand, cells treated with 10 and 30 µM of luteolin revealed strong staining for GPER with a significant increase in MFI to 0.082 and 0.111, respectively. No apparent changes were noticed in MFI following treatment with 100 µM. As a selective agonist for GPER, G-1 led to an obvious increase in the immunostaining of the receptor with 0.078 MFI.

### 3.6. Western Blot

**Figure 7:**
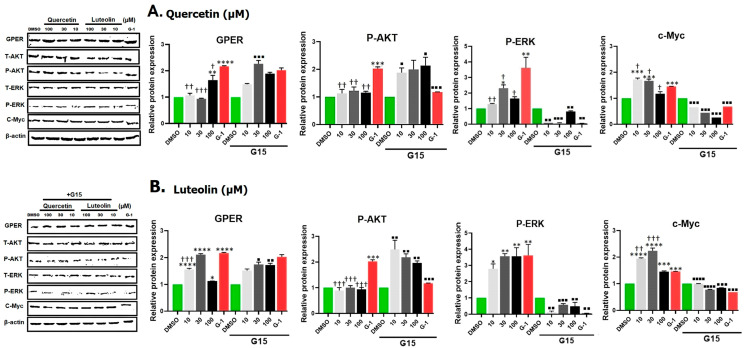
Effects of different treatments with quercetin (**A**) or luteolin (**B**), G-1 (positive control), and 0.2% DMSO (negative control) on the expression of GPER, P-Akt, P-ERK, and c-Myc in A375 CM cell line. Representative Western blot images are shown (left panels). Densitometry analysis was performed and the relative protein expression was normalized to β-actin (right panels). Statistical significance was determined using one-way ANOVA followed by Dunnett’s multiple comparisons test. (* *p* < 0.05, ** *p* < 0.01, *** *p* < 0.001, **** *p* < 0.0001 compared to DMSO; † *p* < 0.05, †† *p* < 0.01, ††† *p* < 0.001, compared to G-1. In the presence of 3 µM of the antagonist G15, the relative protein expressions were significantly reversed where: ■ *p* < 0.05, ■■ *p* < 0.01, ■■■ *p* < 0.001, ■■■■ *p* < 0.0001 compared to the same concentration and ligand in its absence. Densitometry analysis for total Akt, p-Akt/total Akt, total ERK, and p-ERK/total ERK, for treatment with quercetin and luteolin is shown in Supplementary Figure (Figure S1).

Western blot images of the selected target proteins and densitometry analysis after exposure to quercetin or luteolin (10, 30, and 100 µM) and G-1 (1 µM), with or without G15 (3 µM) are shown in Figure 7A,B. Data revealed that the expression of GPER was significantly increased after treatment with G-1. Small concentrations of quercetin caused no increase in GPER but 100 µM of quercetin significantly increased the expression. G15 increased GPER expression in combination with 30 µM quercetin but did not affect the response to other concentrations.

There was an insignificant increase in Akt expression after treatment with quercetin (Figure S1) whereas G-1 reduced the expression but only G-1 was able to notably increase the expression of P-Akt. G15 increased the expression of p-Akt induced by quercetin concentrations but reduced that by G-1.

The expression of total and phosphorylated ERK was higher in quercetin (notably at 30 µM) and in G-1-treated cells, and this was significantly reduced following G15 pretreatment. Expression of c-Myc protein was also increased after incubation with quercetin (10 and 30 µM) and with G-1. This response was abolished with the antagonist G15.

A significant increase in the expression of GPER was detected upon exposure to luteolin and G15 reversed these effects. The expression of Akt was not affected by 10 µM luteolin, unlike 30 and 100 µM, which significantly decreased Akt expression. No significant differences in the expression of P-Akt were observed between luteolin-treated cells and DMSO-treated cells. Pretreatment with G15 significantly reversed the effects of luteolin.

The expression of ERK was significantly increased following exposure to 30 µM of luteolin, while it was significantly reduced at 100 µM of luteolin. On the other hand, luteolin significantly upregulated the expression of P-ERK. These responses were reversed in the presence of G15. High c-Myc expression was observed in luteolin-treated cells compared to DMSO and pretreatment with G15 significantly reduced the expression.

## 4. Discussion

Melanoma, a type of skin cancer, has been traditionally considered a non-hormone-related malignancy. However, emerging evidence suggests that sex hormones, particularly estradiol (E2), might influence melanoma development and progression. One significant discovery in this context was the G-protein coupled estrogen receptor (GPER). GPER was identified as a receptor for estrogens and has been increasingly explored in cancer research, including CM. CM is characterized by its aggressive growth and resistance to conventional treatments, making the exploration of novel therapeutic targets, like GPER, increasingly important.

In a study by Ribeiro et al. (2017), the inhibitory effects of GPER activation on melanoma cells were explored using the mouse K1735-M2 melanoma cell line. Using specific agonists like G-1 and 17β-estradiol, the selective estrogen receptor (ER) modulators tamoxifen (TAM), and its key metabolite endoxifen (EDX) to activate GPER, these authors observed a significant reduction in cell proliferation and cell division, indicating that GPER activation might have tumor-suppressive effects in melanoma cells. Furthermore, they found that the use of GPER agonists modulated MAPK/ERK and PI3K/Akt signaling pathways by reducing the levels of phosphorylated ERK1/2 [40]. These pathways are critical for regulating cell survival, proliferation, and migration, and they are commonly activated in up to 50% of CM, contributing to its aggressive nature and resistance to conventional therapies [41].

The findings of Ribeiro et al. [40] that specific agonists of GPER reduce cell division and proliferation were confirmed by the finding that the specific agonist LNS8801 activated GPER and disrupted the cell cycle, thus causing mitotic arrest that prevented the cells from progressing through critical stages of division, effectively halting their proliferation, and triggering apoptosis [42]. Moreover, the activation of GPER by LNS8801 was found to interfere with key signaling pathways that regulate cell cycle progression and survival, including the MAPK/ERK and PI3K/Akt pathways. These pathways are often deregulated in cancer and contribute to malignant cell behavior, such as uncontrolled proliferation and resistance to cell death. By targeting GPER with LNS8801, it was hypothesized that the balance of these pathways could be shifted in favor of cell cycle arrest and apoptosis, ultimately inhibiting tumor growth [42].

The current study aimed to investigate the role of GPER in mediating the antiproliferative and antitumor effects of two flavonoids, quercetin and luteolin, in human A375 CM cell lines. These antiproliferative and antitumor effects were observed in cell viability, flow cytometry, cell migration, and cell cycle experiments. We hypothesized that these flavonoids exert their effects primarily through GPER activation, which would then modulate critical signaling pathways such as Ras/Raf/Erk and PI3K/Akt. These pathways are fundamental in regulating essential cellular processes like growth, survival, and migration, all of which are crucial in melanoma progression. To examine the estrogenic properties of quercetin and luteolin, their effects were compared to those evoked by the selective agonist, G-1, which can activate the receptor and induce physiological responses at low concentrations [43,44]. Both flavonoids increased the expression of GPER just like G-1 and these effects seemed to be impacted by GPER blockage by the specific antagonist, G15.

To test our hypothesis, we first evaluated the antiproliferative effects of quercetin and luteolin on A375 CM cells using the MTT assay, a standard method for assessing cell viability. Our results revealed that both quercetin and luteolin significantly inhibited the proliferation of A375 cells. Quercetin exhibited an IC_50_ value of 38.6 µM, while luteolin demonstrated a slightly more potent effect, with an IC_50_ value of 19.5 µM (Figure 1). These findings are consistent with previous studies reporting similar effects of quercetin on other cancer cell types, such as T47D cancer stem cells [45]. In the case of luteolin, our results aligned with those of Fan et al. (2019) and Schomberg et al. (2020) who observed comparable inhibitory effects on A431 and A375 cells, with IC_50_ values of 19 µM and 12.5 µM, respectively. Collectively, these results highlight the potential of quercetin and luteolin as effective agents for targeting CM cells. The consistent potency of luteolin across multiple studies [46,47] further supports its broader applicability as a promising anticancer agent.

However, we observed some inconsistencies among different studies in the IC_50_ values for quercetin (99.6 ± 10 µM in one study [48]), which could be attributed to variations in experimental conditions, particularly the type of cell culture medium used [49]. In our study, we cultured the A375 cells in MEM, whereas the study by Cao et al. (2014) used Dulbecco’s Modified Eagle’s Medium (DMEM) for the same incubation period (48 h) [48]. These differences in medium composition, which can affect nutrient availability and overall cell growth, may explain some of the observed variations in the IC_50_ values for quercetin. Such differences are commonly seen in cell-based assays and further underscore the importance of standardizing experimental conditions when comparing data across different studies.

Our MTT assay results strongly corroborated the significant effects of both quercetin and luteolin on A375 CM cells, providing additional support to the suggestion that these flavonoids hold potential as therapeutic agents for CM treatment. Initially, we used G15 to block GPER and we found that the inhibitory effects of the flavonoids and G-1 on A375 cell viability were prevented, providing evidence that activation of GPER is essential for mediating the antiproliferative effects of quercetin and luteolin (Figure 1). Moreover, both flavonoids at higher doses, as well as G-1 reduced cell growth (Figure 2). We next investigated the effects of quercetin and luteolin on apoptosis and cell cycle regulation. Both flavonoids (30 µM and 100 µM) induced significant apoptosis and necrosis in A375 cells (Figure 3), consistent with previous studies on other cancer models [50,51]. Furthermore, consistent with other reports, quercetin and luteolin arrested the cell cycle at the S and G2/M phases (Figure 4) [52,53]. The GPER agonist G-1 also induced apoptosis and cell cycle arrest, providing additional evidence that GPER activation is crucial for the observed effects of quercetin and luteolin on melanoma cells. In addition to their effects on proliferation, both flavonoids as well as G-1 also demonstrated significant anti-migratory activity in A375 melanoma cells, and these effects were reversed to a large extent by the GPER-specific antagonist G15 (Figure 5). This observation aligns with previous studies indicating that quercetin and luteolin can inhibit CM cell migration. For example, Cao et al. (2015) reported that quercetin suppressed A375 migration by interfering with the hepatocyte growth factor (HGF)/c-Met signaling pathway, a key mediator of cell migration and metastasis [54]. Similarly, Yao et al. (2019) showed that luteolin inhibited matrix metalloproteinases (MMP-2 and MMP-9) through the PI3K/Akt pathway and reduced the migration of A375 [55]. Additionally, Hanaf et al. (2024) demonstrated that treatment of ovarian cell lines, OV90 and OVCAR420 as well as immortalized fallopian tube cell line, FT190 with 1 µM G-1 significantly decreased migration after 12 h [56]. When we used G15 to block GPER, the antimigratory effects induced by quercetin, luteolin, and G-1 were partially abolished, which further supports the hypothesis that GPER activation plays a pivotal role in mediating the antiproliferative and antimigratory actions of quercetin and luteolin (Figure 5).

Immunostaining experiments further confirmed the expression of GPER in A375 cells and revealed that treatment with the agonist, G-1 upregulated GPER expression (Figure 6). Our results are in agreement with Almedia, et al. (2022) who showed that the expression level of GPER in glioblastoma C6 was enhanced following treatment with E2 or G-1 for 48 h [57]. Interestingly, quercetin and luteolin, particularly at 30 µM, resulted in a pronounced increase in immunofluorescence, hence GPER expression, compared to G-1, suggesting that these flavonoids may act as agonists to GPER and may exert their physiological effects through activation of GPER in a way similar to that of G-1. This observation diverges from the findings of Sun et al. (2016), who reported that G-1 did not affect GPER expression in the A375 CM cell line [31], presumably due to differences in experimental conditions, such as the lower concentrations (0.2, 0.4, 0.8 µM) and the shorter incubation period (24 h) used in their study. Of note, at the highest concentration (100 µM), quercetin caused a lower MFI value than DMSO, while the MFI of luteolin was similar to that of DMSO, possibly due to receptor desensitization and internalization as a response to sustained activation by high doses of the agonists [58].

Building on these results, we aimed to investigate the underlying mechanisms through which quercetin and luteolin exerted their antiproliferative effects. We examined the impact of GPER activation by G-1, quercetin, and luteolin on the Ras/Raf/Erk and PI3K/Akt signaling pathways by Western blotting. First, we found that G-1, as a GPER agonist, increased the expression level of GPER in A375 CM cells. Quercetin at 100 µM and luteolin at all concentrations also increased the expression of GPER. We observed some differences between the results obtained by immunofluorescence and Western blot regarding the effect of flavonoids on GPER expression. These differences could be attributed to the shorter stimulation time we used in Western blot (24 h) compared to immunofluorescence assay (48 h). These differences suggested that quercetin at lower doses (10 and 30 µM) may need a longer time to interact with GPER and increase its expression, unlike luteolin which was more efficient in binding to the receptor and elevating its level in a shorter time. ERK protein is the key effector in the Ras-Raf-Erk signaling pathway. Although it was found that about 40% of human cancers were associated with aberrant ERK pathway activity, a recent review by Timofeev and collaborators cited the evidence that BRAF mutant CM, like A375 cells, are sensitive to ERK activation and overexpression of ERK has an inhibitory effect in this cell line [59]. Others suggested that while ERK signaling is typically associated with cell survival, it can also promote cell death under certain conditions. Prolonged or excessive activation of ERK, often induced by stress or DNA damage, can lead to cellular dysfunction and apoptosis [60]. Consistent with the above observations, we observed an increase in the expression of phosphorylated ERK (P-ERK) in response to treatment with both flavonoids as well as in G-1 (Figure 7). Quercetin or luteolin antioxidant properties may then alter the cellular redox state, creating an environment in which ERK activation shifts from a pro-survival signal to one that increases cellular stress, potentially shifting the balance toward cell death. These findings underscore the complexity of signaling networks in CM cells and highlight the balance between pro-survival and pro-apoptotic pathways in response to quercetin and GPER activation. Taken together, the data indicate that GPER activation can transactivate the epidermal growth factor receptor (EGFR) and activate the Ras/Raf/Erk pathway. This conclusion is substantiated by the finding that the effect of both flavonoids and G-1 was consistently reversed in the presence of the antagonist G-15.

c-Myc is a transcription factor that plays an important role in proliferation, metabolism, cell cycle, and apoptosis. Its expression is elevated in about 50% of cancers including CM, mainly through Ras/Raf/Erk and/or PI3K/Akt pathways, leading to tumor progression [61]. In the current study, we noticed an unexpected increase in the level of c-Myc following treatment with both flavonoids and G-1. This observation was different from the findings of Natale et al. (2017), who showed that loss of c-Myc is a major pathway of the anti-proliferative effects of GPER signaling in CM [62]. Although c-Myc is a potent stimulus of growth and proliferation, it was found to sensitize cells to apoptosis, which limits its tumorigenic capacity. This c-Myc sensitization was interpreted as triggered by the limitation of the tricarboxylic cycle [63] or due to an imbalance of metabolic/energetic supply and demand. We speculate that the observed upregulation of c-Myc may represent a compensatory mechanism in response to DNA damage or toxicity induced by the treatments. Alternatively, it was proposed that c-Myc directs P53 functions towards apoptosis rather than cell cycle arrest and reduces the expression of anti-apoptotic proteins such as Bcl-xL and Bcl-2 [64]. Within the same context, it was previously demonstrated that c-Myc upregulation leads to the accumulation of P53, which increases cell apoptosis in murine intestinal enterocytes following DNA damage [65]. The increase in the expression of c-Myc by both flavonoids and by G-1 was significantly reversed when cells were treated with the G-15 antagonist, indicating that this increase in c-Myc was mediating GPER activation by these phytoestrogens, and again confirming the potential therapeutic effect of these flavonoids in management of CM.

We also observed no change in Akt phosphorylation (P-Akt) after quercetin and an insignificant decrease after luteolin but an unexpected increase after G-1 treatment, which contrasts with the findings of Liu et al. (2022), who reported a decrease in P-Akt following G-1 treatment in SKBR-3 breast cancer cells [66]. This difference may be attributed to differences in cell type and experimental conditions. High levels of P-Akt are usually associated with increased cell growth and anti-apoptotic effects. Additionally, although quercetin and luteolin did not significantly alter Akt phosphorylation in A375 cells, luteolin did reduce total Akt levels (Figure S1), in agreement with the findings by Yao et al. (2019) in melanoma cells, suggesting an inhibition of cell proliferation and survival [55]. These results suggest that GPER-mediated effects on the PI3K/Akt pathway may vary depending on the cellular context, underscoring the complexity of GPER signaling in CM. In our experiment, the antagonist G15 significantly reversed the effects of the flavonoids as well as those of G-1, indicating that these effects were mediated through GPER activation. Concerning the estrogen agonistic potency of quercetin and luteolin, Nordeen et al. (2013) showed that luteolin displayed greater estrogen agonistic activity than quercetin in T47D (A1-2) cells that express progesterone and estrogen receptors [67]. Little differences were noticed in our study between the effects of the two phytoestrogens on the A375 CM cell line. As we noticed that 100 µM could be more toxic to cells, the dose of 30 µM is preferable in future research on this cell line, since it is also the nearest to the IC_50_ of quercetin and luteolin. The structure of the two flavonoids plays a fundamental role in the nature and potency of their activity. The number and position of hydroxyl groups, as well as the presence of the double bond between carbons 2 and 3 and the presence or absence of methyl groups, are the main factors that determine the estrogenic activity of these flavonoids [68,69]. Quercetin and luteolin have five and four hydroxyl groups, respectively, and both have a double bond between C2–C3 that confers on them high potency.

Our study provides novel insights into the role of GPER in mediating the anticancer effects of quercetin and luteolin in CM cells. To the best of our knowledge, this is the first study to demonstrate that these flavonoids can induce antiproliferative, pro-apoptotic, and antimigratory effects through GPER activation in A375 CM cells, highlighting the potential of GPER-targeted therapeutic strategies for CM treatment. Future studies should continue to explore the molecular mechanisms underlying the role of GPER in melanoma and other cancers, with a particular focus on the differential cellular responses to phytoestrogens like quercetin and luteolin. Understanding these mechanisms will be essential for developing more effective therapeutic approaches targeting GPER in melanoma and other malignancies.

There are some limitations associated with the present study. While the in vitro model used in this study provides a controlled environment that allows for the study of specific cellular and molecular mechanisms, it does not capture the complexity of the whole organism. It lacks the intricate biological interactions between cell types, the immune system, and tissue-specific factors present in vivo. Recently, researchers have suggested the use of 3-D cell culture [34] rather than the 2-D culture we used to better imitate the in vivo systems. Also, incorporating quantitative PCR (qPCR) to assess gene expression would provide complementary insights into the transcriptional regulation of the proteins of interest. While Western blotting is a widely used and reliable method for detecting protein abundance, it does not provide insights into transcriptional regulation or gene expression levels. Furthermore, it is a semi-quantitative technique, subject to variations in sample preparation, antibody specificity, and detection methods, which can affect accuracy and reproducibility. Another limitation is the lack of assessment of anoikis or apoptosis-related proteins to help understand the underlying mechanisms involved in flavonoid-induced cell death. Finally, due to logistic reasons, some of the experiments in this study did not incorporate the use of the specific GPER antagonist G15.

## 5. Conclusions

The present study provides evidence that the phytoestrogens quercetin and luteolin exert antitumor effects on the A375 CM cell line, at least in part, through the activation of GPER. The two flavonoids inhibited the proliferation by enhancing apoptosis and blocking the cell cycle at the S and G2/M phases. The study also revealed that quercetin and luteolin reduced the migration ability of CM cells. Additionally, the present work showed that modulating the expression of P-ERK protein, which is the main effector in the Ras/Raf/Erk pathway along with the transcription factor, c-Myc might be responsible for the antitumor effects of quercetin and luteolin in CM cell. Our findings suggested that quercetin and luteolin could be further developed to be potential anti-CM agents through the activation of GPER.

## Figures and Tables

**Table 1:** Primary antibodies that were used in Western blots.

Antibody	Host Species	Cat. No.	Supplier	Dilution	Band Size (kDa)
GPER	Rabbit	ES11471	ELK Biotechnology	1:500	41
ERK1/2	Rabbit	EA331	ELK Biotechnology	1:500	42–44
P-ERK1/2	Mouse	Sc-136521	Santa Cruz	1:500	42–44
Akt 1/2/3	Mouse	Sc-56878	Santa Cruz	1:500	62
P-Akt	Rabbit	Ab38449	Abcam	1:500	56
c-Myc	Rabbit	EA053	ELK Biotechnology	1:1000	57–65
B-actin	Rabbit	GW0061R	GenoChem World	1:1000	42

## Data Availability

The original contributions presented in this study are included in the article and in the Supplementary File. Further inquiries can be directed to the corresponding author.

## Supplementary Materials

The following supporting information can be downloaded at: https://www.mdpi.com/article/10.3390/life15030417/s1, Figure S1: Expression of the proteins T-AKT, P-AKT, T-ERK, P-ERK and their ratios when treated with quercetin or luteolin in the absence or the presence of G15.
