# Supplementary material for: Antitumor Effects of Quercetin and Luteolin in A375 Cutaneous Melanoma Cell Line Are Mediated by Upregulation of P-ERK, c-Myc, and the Upstream GPER
Source: Life (Basel). 2025 Mar 7;15(3):417. doi: 10.3390/life15030417 (PMC11943993; doi:10.3390/life15030417)
Supplement: Supplementary file 1 [file life-15-00417-s001.zip › life-3488072-supplementary.pdf]

Supplementary Material

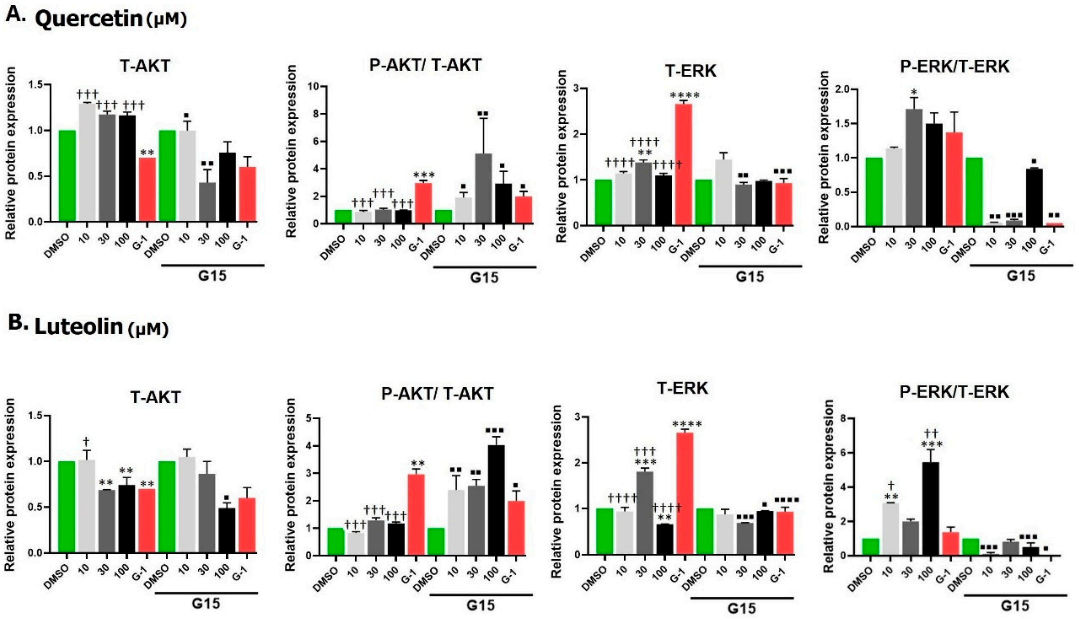

Figure S1. Effect of quercetin (**A**) and luteolin (**B**), G-1 (positive control), and 0.2% DMSO (negative control) on the protein expression of T-AKT, P-AKT/T-AKT, T-ERK, and P-ERK/T-ERK, in A375 cells, assessed by Western blotting. Statistical significance was determined using one-way ANOVA followed by Dunnett's multiple comparisons test. (*$p < 0.05$, **$p < 0.01$, ***$p < 0.001$, ****$p < 0.0001$ compared to DMSO; †$p < 0.05$, ††$p < 0.01$, †††$p < 0.001$, ††††$p < 0.0001$ compared to G-1). In the presence of 3 μM of the antagonist G15, the relative protein expressions were significantly reversed, with the following levels of significance: ■$p < 0.05$, ■■$p < 0.01$, ■■■$p < 0.001$, ■■■■$p < 0.0001$ compared to the same concentration and ligand in the absence of G15.
